# Non-coaxiality of sand under bi-directional shear loading

**DOI:** 10.1098/rsos.172076

**Published:** 2018-05-16

**Authors:** Yao Li, Yunming Yang

**Affiliations:** 1 School of Highway, Chang'an University, Xi'an, People's Republic of China; 2 International Doctoral Innovation Centre, Department of Civil Engineering, The University of Nottingham, Ningbo, People's Republic of China

**Keywords:** non-coaxiality, simple shear test, sand

## Abstract

This study aims to investigate the effect of consolidation shear stress magnitude on the shear behaviour and non-coaxiality of soils. In previous drained bi-directional simple shear test on Leighton Buzzard sand, it is showed that the level of non-coaxiality, which is indicated by the angle difference between the principal axes of stresses and the corresponding principal axes of strain rate tensors, is increased by increasing angle difference between the direction of consolidation shear stress and secondary shearing. This paper further investigated the relation and includes results with higher consolidation shear stresses. Results agree with the previous relation, and further showed that increasing consolidation shear stresses decreased the level of non-coaxiality in tests with angle difference between 0° and 90°, and increased the level of non-coaxiality in tests with angle difference between 90° and 180°.

## 1. Introduction

In experiment and numerical modelling, it is showed that the principal axes of stresses and the corresponding principal axes of strain rate tensors often do not coincide [1–4]. In most of numerical modelling soil models, these axes are considered as generally coincident, which neglects the non-coaxiality and may lead to unsafe simulation results [5–10].

Due to the limitation of testing apparatus, the non-coaxiality is not well understood, especially when shear stresses exist. In tests conducted by Li *et al*. [11] using the first commercially available variable direction dynamic cyclic simple shear system (VDDCSS), a procedure is introduced in determining the rotation angle of principal axes of stresses and plastic strain rate using data obtained from a typical bi-directional simple shear test. The level of non-coaxiality can be determined by the angle difference between the two determined angles. In the procedure, two methods are considered for bi-directional simple shear test, the first one considers shear stress in the secondary shearing direction as shear stress in calculation, and the second method considers total shear stress (obtained by combining the components in the *x* and *y* directions) as shear stress in calculation. Results obtained from the two methods showed a good agreement on the change of non-coaxiality. In tests conducted by Li *et al.* [11], tests with different relative densities, vertical stress and angle difference between consolidation shear stress and secondary shear stress were considered. Results showed that the level of non-coaxiality is decreased by the increasing relative density, decreasing vertical stress and decreasing angle difference. However, in tests conducted by Li *et al*. [11], only one consolidation shear stress ratio (CSR = 0.1) is considered and the magnitude of consolidation shear stress is neglected, which showed its significant effect on similar studies.

This study includes test results that consider a higher consolidation shear stress ratio (0.2), and the effect of the magnitude of consolidation shear stress on non-coaxiality is determined by comparing the two groups of tests that have different consolidation shear stress ratios (magnitudes).

## 2. Methodology

The same testing device (VDDCSS), testing material (Leighton Buzzard sand Fraction B), sample reconstitution method (dry deposition technique) and testing method are used in this study as those used by Li *et al*. [11–14]. Dry samples were prepared with the relative density of 68%. It should be noted that only dry specimens can be tested in the testing apparatus due to the difficulties in saturation. Testing details are introduced by Li *et al*. [11–13].

**Figure 1.**
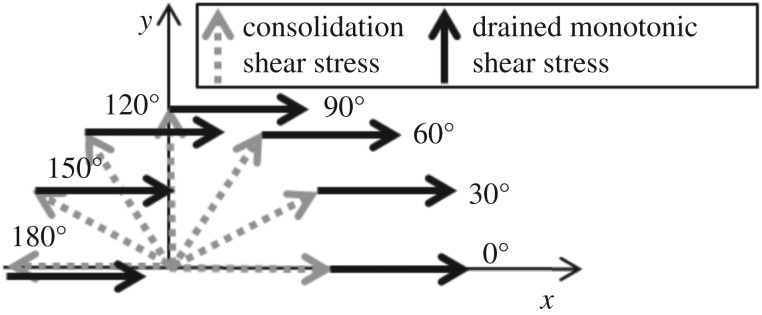
Stress paths of tests with different stress histories [11].

In this study, samples are first consolidated under a vertical stress of 200 kPa and a shear stress of 40 kPa in various directions. The direction varies from 0° to 180° with the interval of 30°. Figure 1 shows the stress path of performed tests.

## 3. Results

**Figure 2.**
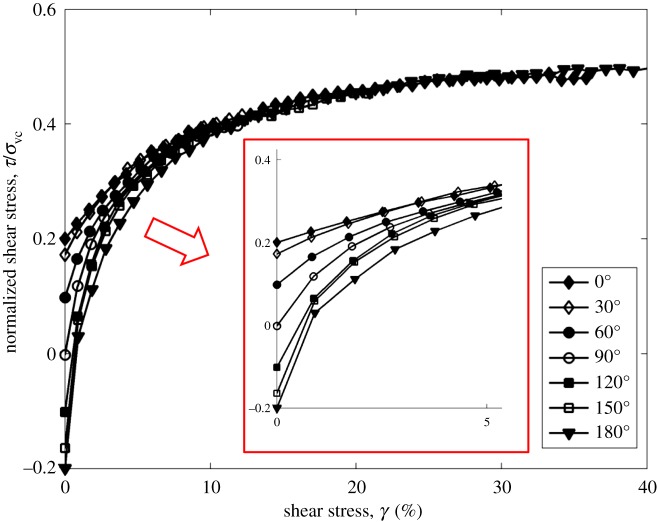
Development of the normalized shear stress in different stress paths at CSR = 0.2.

Results show that, in the tests with the angles of 30°, 60°, 90°, 120° and 150°, failure happens in the *y* axis at the shear strain around 10%. Figure 2 shows the development of normalized shear stress in different stress paths before failure. The stress–strain behaviours between tests with different stress paths are similar to those in tests at CSR = 0.1 [11].

**Figure 3.**
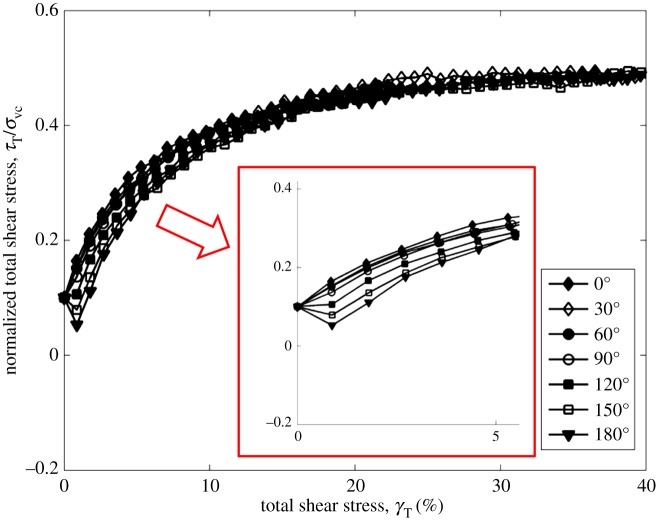
Development of the normalized total shear stress in different stress paths at CSR = 0.1 [11].

**Figure 4.**
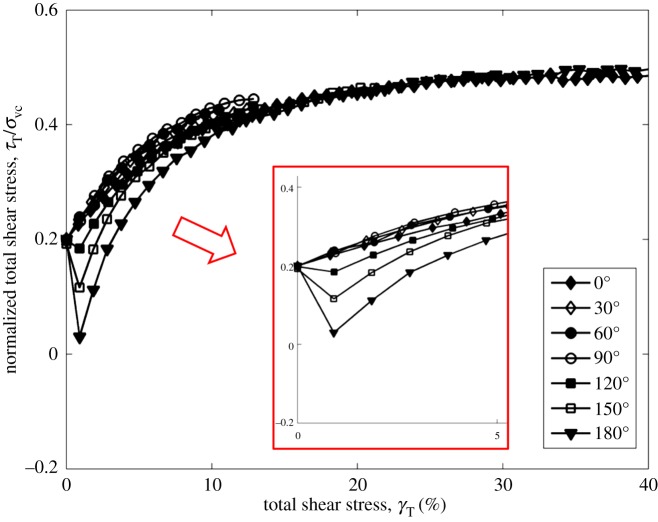
Development of the normalized total shear stress in different stress paths at CSR = 0.2.

Figures 3 and 4 show the normalized total shear stress development in different stress paths before failure at the CSR = 0.1 and 0.2. It can be seen that the development of total shear stress among tests with different stress paths is different from those in tests at CSR = 0.1. In tests at CSR = 0.1, the 0° test has the greatest total shear stress at small shear strain, followed by the 30°, 60° and 90° tests. In tests at CSR = 0.2, the test with the angle of 90° has the greatest total shear stress at small shear strain, followed by the 60°, 30° and 0° tests. It should be noted that the increasing magnitude of CSR increases the constant shear stress in the *y* direction during shear, especially in the 60° and 90° tests. As a result, the 60° and 90° tests have the greatest total shear stress at the same shear strain when considering the shear stress in the *y* direction.

**Figure 5.**
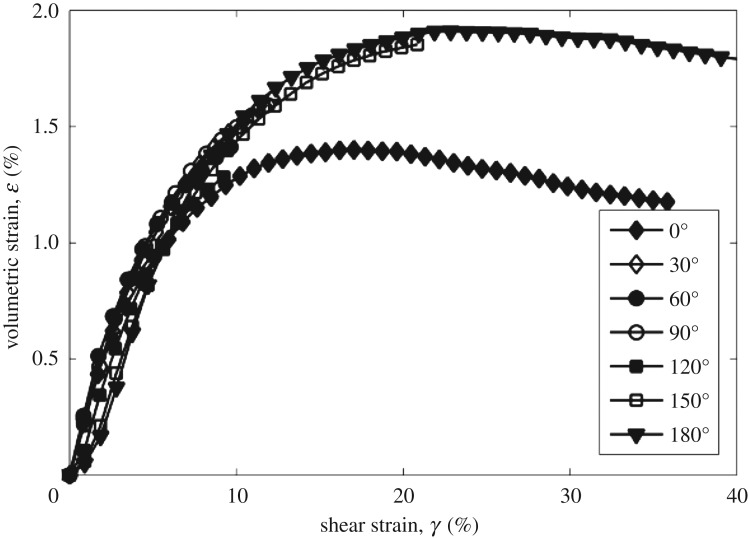
Development of volumetric strain in different stress paths at CSR = 0.2.

**Figure 6.**
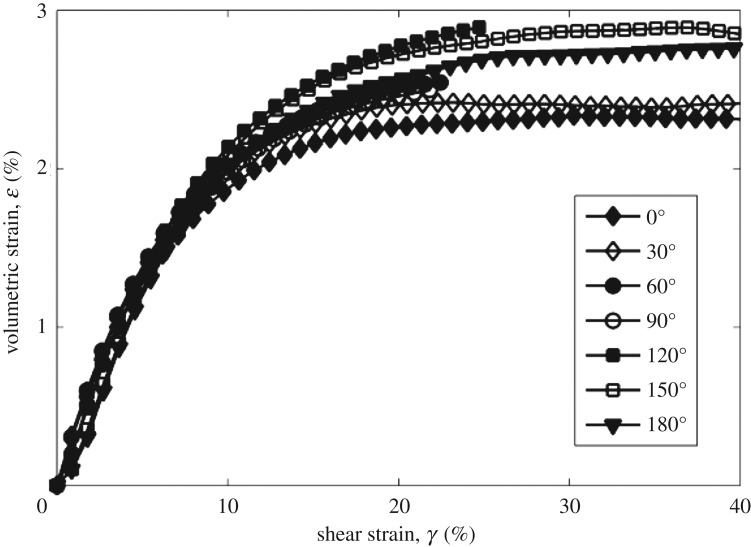
Development of the volumetric strain in different stress paths at CSR = 0.1 [11].

Figure 5 shows the development of volumetric strain in different stress paths at CSR = 0.2. It should be noted that most tests failed at the early stage of shearing, and shows little difference in the development of volumetric strain at the beginning of shear. The tests with the angles of 0° and 180° develop greater shear strains, and corresponding volumetric strains are well recorded. It shows that the test with the angle of 0° reached the peak volumetric strain first. Similar behaviour is observed in the test with the angle of 180°, and it reaches the peak volumetric strain at a higher shear strain. The peak volumetric strain of the test with the angle of 180° is greater than that in the test with the angle of 0°, which shows a similar trend to that in the tests at CSR = 0.1. In addition, tests at CSR = 0.2 show a greater dilative behaviour than the tests at CSR = 0.1, as shown in figures 5 and 6.

**Figure 7.**
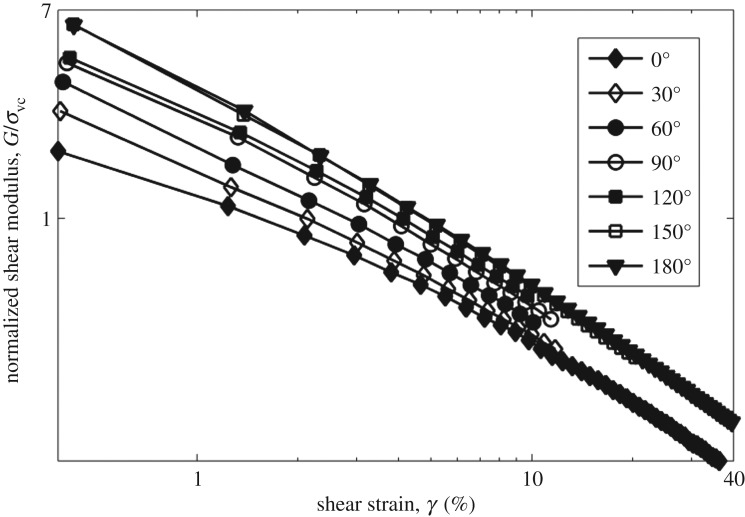
Normalized shear modulus versus shear strain in tests with different stress paths under the CSR = 0.2.

**Figure 8.**
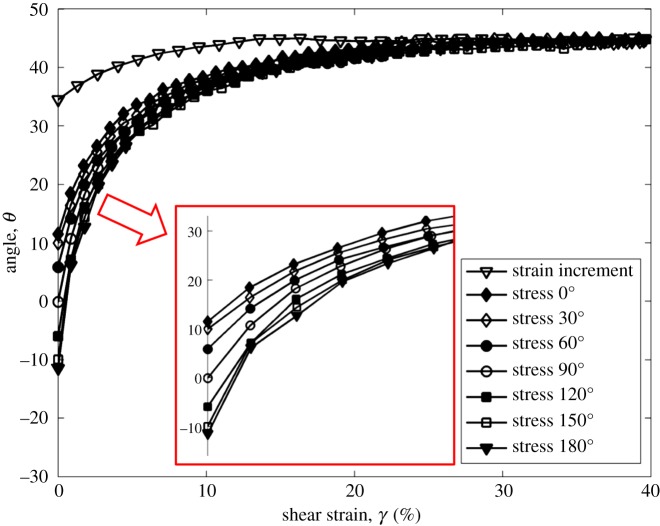
Rotation of principal axes of stresses and strain increment in different stress paths at CSR = 0.1 [11].

**Figure 9.**
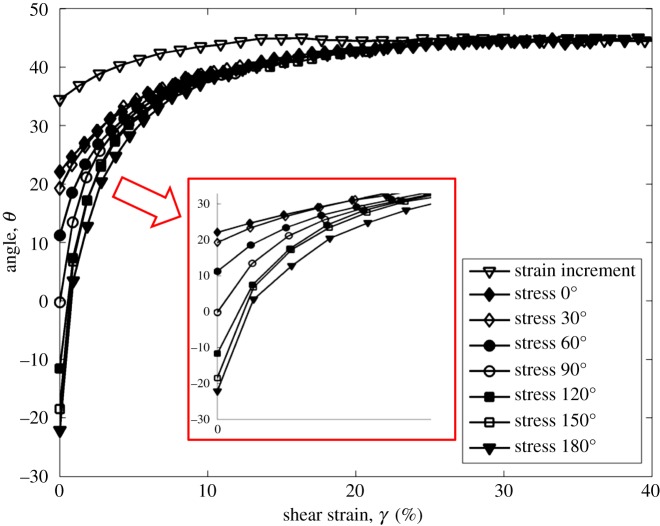
Rotation of principal axes of stresses and strain increment in different stress paths at CSR = 0.2.

**Figure 10.**
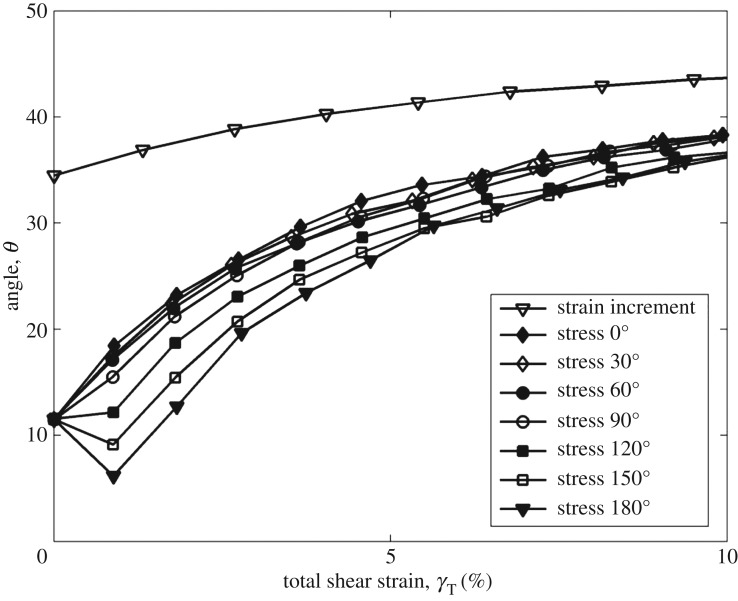
Rotation of principal axes of total stresses and total strain increment in different stress paths at CSR = 0.1 [11].

**Figure 11.**
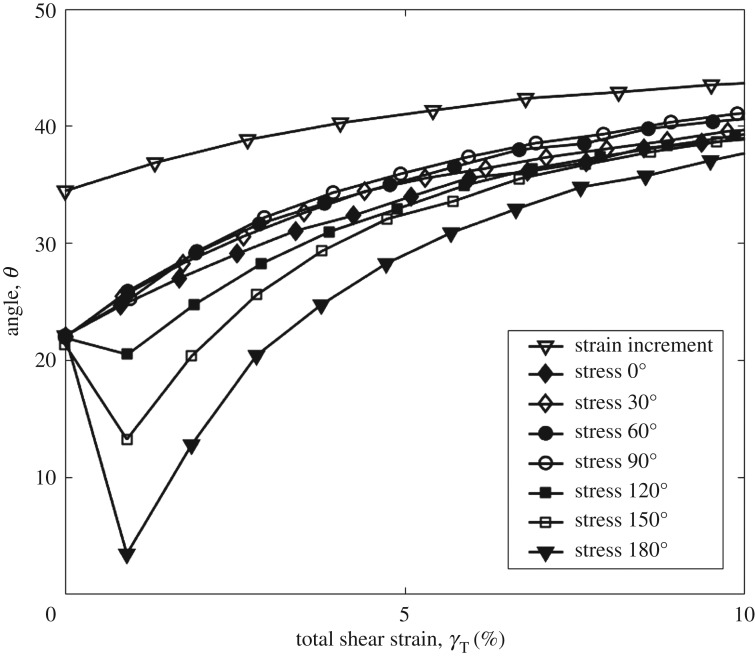
Rotation of principal axes of total stresses and total strain increment in different stress paths at CSR = 0.2.

Figure 7 plots the log of normalized shear modulus versus the log of shear strain at CSR = 0.2. Shear modulus is increased by the increasing angle, and the trend is clearer than that in tests with the CSR = 0.1. Figures 8 and 9 show the rotation of principal axes of stresses and strain increment in different stress paths at CSR = 0.1 and 0.2. The trend among different angles is similar, in which the increasing angle increases the non-coaxiality at the beginning of shearing, and the non-coaxiality decreases during shear. However, the level of non-coaxiality is different from tests at CSR = 0.1. In the tests with the angle of 0°, 30° and 60°, the level of non-coaxiality is decreased by increasing CSR, while the level of non-coaxiality is increased in the tests with the angle of 120°, 150° and 180°. Figures 10 and 11 show the rotation of principal axes of total stresses and total strain increments in different stress paths at CSR = 0.1 and 0.2. It can be seen that the 0° test has the greatest total shear stress at small shear strain in tests at CSR = 0.1, while the test with the angle of 90° has the greatest total shear stress at small shear strain in tests at CSR = 0.2. This finding indicates the importance of considering total shear stress in determining the angle difference on non-coaxiality. In addition, it is interesting to note that, although the level of non-coaxiality is changed by increasing CSR, the principal axes of stresses and strain increment coincide at the same shear strain in tests with different CSRs.

## 4. Conclusion

This study conducted a series of bi-directional simple shear tests considering a consolidation shear stress ratio of 0.2 which is greater than that in the study conducted by Li *et al*. [11]. By comparing the obtained result in different levels of consolidation shear stress, the effect of consolidation shear stress ratio on non-coaxiality is found. In addition, the effect of angle difference between consolidation shear stress and secondary shear stress on non-coaxiality is updated.

1. The level of non-coaxiality can be either increased or decreased by the increasing CSR. Specifically, when the angle between consolidation shear stress and drained shear stress is small (e.g. 0°–90°), the level of non-coaxiality is decreased by the incresing CSR. When the angle is large (e.g. 90°–180°), the level of non-coaxiality is increased by the increasing CSR. This finding shows that the angle difference and magnitude of CSR have a combined effect on non-coaxiality.
2. The effect of angle difference between consolidation shear stress and secondary shear stress on non-coaxiality generally agree with that in tests at CSR = 0.1. However, in results considering total shear stress as shear stress in calculation, in tests at CSR = 0.1, the 0° test has the greatest total shear stress at small shear strain, and in tests at CSR = 0.2, the test with the angle of 90° has the greatest total shear stress at small shear strain. This finding indicates the importance of considering total shear stress in determining the angle difference on non-coaxiality.

Non-coaxiality has been widely considered in constitutive models of soils, and many constitutive models of soils are developed using hollow cylinder tests. This study used bi-directional simple shear tests which can better replicate *in situ* conditions, and expermental data and result could improve soil constitutive models which help simulate real shear behaviour of soils under complex stress environment.

## Data accessibility

Data are deposited at Dryad (http://dx.doi.org/10.5061/dryad.pp2t3) [15].
